# The Hospital World

**Published:** 1910-12-10

**Authors:** 


### The Hospital World.

### COMPLIMENTARY BANQUET AND PRESENTATION TO MR. C. J. BOND, F.R.C.S

Mr. C. J. Bond, F.R.C.S., senior surgeon to the Leicester Infirmary, was entertained to a complimentary banquet last week by the members of the medical profession of the district, to mark his retirement from his private consulting practice.

Dr. C. F. Bryan, the senior member of the local medical Societies, presided over a large gathering of medical men of Leicestershire and the surrounding counties, and, in proposing the health of their guest, remarked how Mr. Bond, during his twenty-five years' work among them, had endeared himself to them all and won their esteem. His kindliness of manner, professional skill, and the complete confidence they had in him they desired to express publicly by this dinner.

At a reception later a presentation of silver plate was made to Mr. Bond by Dr. Bryan, who made an appreciative reference to his work and career. Educated at Repton, he entered University College in 1875, where he gained great distinction in his medical studies, and was awarded gold medals in anatomy and physiology and silver medals in anatomy, surgery, midwifery, and medical jurisprudence. After qualification, he became assistant demonstrator of anatomy to his hospital, and was subsequently appointed house surgeon to the Bedford County Infirmary. In 1883 he was appointed house surgeon to the Leicester Infirmary, which post he filled to the greatest advantage of the institution and to the entire approval of the Board of Governors till 1886. He was then appointed hon. assistant surgeon to the institution, and, in 1886, after being admitted a Fellow of the Royal College of Surgeons of England, he was appointed honorary surgeon, and at the present time he was the senior surgeon. Besides this, he was an honorary colonel of the R.A.M.C. (T.), an ex-President of the Midland Branch of the British Medical Association, a member of the Leicester Medical Society, a President of the local Literary and Philosophical Society, a trustee of Sir Thomas White's Charity, a member of the Museum and Art Committee of the Town Council, a medical officer of the C.O.S. and other Societies of the town. His work for the British Medical Association was well known. When in 1905 the Association held its meetings in Leicester Mr. Bond delivered a most instructive address on "Surgery," and in the following year he opened a discussion on septic peritonitis at Toronto, where he was greatly honoured and enthusiastically received. His contributions to medical and surgical literature were numerous and widely known. As a consulting surgeon Mr. Bond had endeared himself to the profession and public alike, and while they congratulated him on his well-earned rest they would regret to lose his valuable experience in their work. Though his retirement would be a great loss to the district, it was a source of general satisfaction that he would continue his valuable services to the Leicester Infirmary and to devote a great deal of his leisure to scientific research, which was of powerful fascination to him, and in which he had achieved considerable fame.

Dr. Bryan then presented a silver tea and coffee service to Mr. Bond as a memento of the esteem in which he was held by the medical profession of the town and county.

Mr. Bond feelingly acknowledged the great honour which his colleagues had paid to him, and the many kindnesses which they had shown him.

On the silver tray was the following inscription : "To C. J. Bond, F.R.C.S., from his medical friends in Leicester and the neighbourhood, as a token of affection and esteem on his retirement from practice, 1910." Each piece of the silver tea and coffee service was engraved with the monogram "C. J. B."

### Personalia.

It is not only the House of Commons which loses a distinguished and respected gentleman by the death of Mr. J. E. Ellis. Mr. Ellis was a Quaker, and his education at the Friends' School at Kendal perhaps fostered that public spirit and philanthropy which found expression later on in his work for charitable institutions. The Children's Hospital and the Sanatorium in Sherwood Forest found in him a warm and generous supporter, and the General Hospital, Nottingham, was proud to include him in its list of life governors. The coveted distinction of Privy Councillor was perhaps the crown of his Parliamentary honours ; but this did not distract his attention from social work, and the hospital at Nottingham continued till the end of his life to receive generous encouragement and support from him. It is typical of the confidence with which he filled others that the Rushcliffe Division of Nottinghamshire returned him as its member for an uninterrupted period of twenty-five years. He held office in 1905 as Under-Secretary for India.

The eighth annual dinner of the Incorporated Association of Hospital Officers, held a week ago under the presidency of Mr. Walter Alvey, was a most successful gathering. Sir Savile Crossley, Bart., and Lady Crossley were the guests of the evening, and among those present were Mr. P. Michelli, Lord Claud Hamilton, M.P., Major Tyler, Mr. Eason (Manchester), Mr. W. Carnt, Mr. and Mrs. Johnson, and Messrs. Arnaudin, Carrol, Mackay, and Thomas. Mr. Sydney Phillips proposed "The Hospitals," coupling this with the name of Sir Savile Crossley, and remarking that the interests of the voluntary hospitals were safe in the hands of the secretaries of the King's Fund. Sir Savile, in response, said the fund with which he was connected had progressed from year to year, and up to the present year, when King George was graciously pleased to give his patronage to the fund, they had exceeded the amount ever contributed, compared with corresponding months of past years, by over £3,000. When the fund was first started it was said that it was not wanted, and that it would get in the way; but he had always believed in a central body, which would control the purse-strings to some extent. It enabled the very best advice to be procured, and everyone would agree that they had been very fortunate in the men they had obtained. A central fund could be of assistance in the collecting of useful information on points of common interest, and with regard to a central committee of inquiry. The inquiry, for instance, as to the relations between hospitals and medical schools had been a very useful one, and possibly there might be another into the abuse or non-abuse of the out-patient system. Although in the future expenses might be greater, and numerous as were the forms of the Chancellor of the Exchequer, the time was a good one for appealing to the public, and if the action of the King in taking up the fund was brought home to the public of London at large more sympathy for the suffering poor and a greater desire to benefit them would be the result. Lord Claud Hamilton, M.P., who replied to the toast of "The Visitors," which was felicitously proposed by Mr. H. H. Jennings, said that outside criticisms against hospitals often had the effect of directing public attention to appeals which would be otherwise overlooked.

Viscount Kitchener of Khartoum has been appointed a vice-president of the Middlesex Hospital.

The death of Mr. H. D. Marshall, who was for many years a trustee and an active worker for the Driffield Cottage Hospital, near Hull, is mentioned with just regret in the annual report. Not only was Mr. Marshall a trustee, but he took an active part in the routine work, which less enthusiastic persons shun sometimes, by sitting as a regular member of the executive committee. A cottage hospital provides plenty of work and opportunity to a keen committeeman, and the institution at Driffield is no exception to this rule.

### Elections and Appointments.

Mr. John Dunn, Mr. T. R. J. Logan, Mr. D. McKechnie, and Mr. George A. Macbeth have been re-elected Governors of the Victoria Infirmary, Glasgow.

Councillor Horrell has been elected chairman of the Isle of Thanet Joint Hospital Board. The previous chairman, whose term of office is just over, was Alderman Clutton, ex-Mayor of Ramsgate.

Mr. H. E. Allanson, M.B., Ch.B.(Vict.), has been appointed house physician to the Ancoats Hospital, Manchester, and Mr. Niel McDonald, M.B., Ch.B.(Vict.), resident casualty officer and anæsthetist.

The officers of the Driffield Cottage Hospital, near Hull, have been elected as follows : President, Sir Tatton Sykes, Bart. ; vice-presidents, Sir Luke White, Mr. H. Holt, Mr. H. Campbell, Mr. Prince Smith, and Colonel Duncombe. Mr. W. G. Hives has been re-elected hon. secretary and treasurer, and a high compliment was paid to him for his work.

At the monthly meeting of the Board of Management of Ancoats Hospital, Manchester, William Armitage, Esq., was elected chairman of the Board, the vacancy caused by the death of S. L. Helm, Esq. ; J. R. Oliver, Esq., was elected hon. treasurer and chairman of the house committee ; Alfred E. Goddum, Esq., was elected hon. secretary.

